# Mitochondrial Oxidative Stress Significantly Influences Atherogenic Risk and Cytokine-Induced Oxidant Production

**DOI:** 10.1289/ehp.1002857

**Published:** 2010-12-17

**Authors:** Corey M. Harrison, Melissa Pompilius, Kent E. Pinkerton, Scott W. Ballinger

**Affiliations:** 1 Department of Pathology, Division of Molecular and Cellular Pathology, University of Alabama–Birmingham, Birmingham, Alabama, USA; 2 Institute of Toxicology and Environmental Health, University of California–Davis, Davis, California, USA; 3 Department of Environmental Health, University of Alabama–Birmingham, Birmingham, Alabama, USA

**Keywords:** atherogenesis, mitochondria, oxidant stress, SOD2, tobacco smoke

## Abstract

**Background:**

Oxidative stress associated with cardiovascular disease (CVD) risk factors contributes to disease development. However, less is known whether specific subcellular components play a role in disease susceptibility. In this regard, it has been previously reported that vascular mitochondrial damage and dysfunction are associated with atherosclerosis. However, no studies have determined whether altered mitochondrial oxidant production directly influences atherogenic susceptibility and response in primary cells to atherogenic factors such as tumor necrosis factor-α (TNF-α).

**Objectives:**

We undertook this study to determine whether increased mitochondrial oxidant production affects atherosclerotic lesion development associated with CVD risk factor exposure and endothelial cell response to TNF-α.

**Methods:**

We assessed atherosclerotic lesion formation, oxidant stress, and mitochondrial DNA damage in male apolipoprotein E (apoE)-null mice with normal and decreased levels of mitochondrial superoxide dismutase-2 (SOD2; apoE^−/ −^ and apoE^−/ −^, SOD2^+/−^, respectively) exposed to environmental tobacco smoke or filtered air.

**Results:**

Atherogenesis, oxidative stress, and mitochondrial damage were significantly higher in apoE^−/ −^, SOD2^+/−^ mice than in apoE^−/ −^ controls. Furthermore, experiments with small interfering RNA in endothelial cells revealed that decreased SOD2 activity increased TNF-α–mediated cellular oxidant levels compared with controls.

**Conclusions:**

Endogenous mitochondrial oxidative stress is an important CVD risk factor that can modulate atherogenesis and cytokine-induced endothelial cell oxidant generation. Consequently, CVD risk factors that induce mitochondrial damage alter cellular response to endogenous atherogenic factors, increasing disease susceptibility.

Cardiovascular disease (CVD) is the leading cause of morbidity and mortality in the Western world (Rosamond et al. 2007). Atherosclerosis, a chronic inflammatory disease that results in the deposition of oxidized lipoproteins that form fatty plaques, accounts for nearly 75% all deaths from CVD (Rosamond et al. 2007). Although the underlying etiology of CVD development is still somewhat controversial, there is a general consensus that atherosclerotic lesions result from oxidative stress associated with CVD risk factors. Among these factors, hypercholesterolemia and/or tobacco smoke exposure are strong predictors of CVD development. Clinical and epidemiological studies have clearly shown that increased low-density lipoprotein (LDL) cholesterol levels are associated with increased atherogenic risk, and subsequent studies have revealed that LDL must be oxidatively modified to damage the arterial wall and endothelium (Liao et al. 1995; Napoli et al. 2000), consistent with the notion that oxidative stress plays a key role in endothelial dysfunction and atherogenesis (Freeman et al. 1995; Ignarro et al. 1999). Similarly, tobacco smoke exposure was identified as a major atherogenic risk factor in the Framingham heart study (Seltzer 1989), and epidemiological evidence has unequivocally confirmed that smoking is a major CVD risk factor (Glantz and Parmley 1995). Clinical data from additional studies demonstrate that both active and passive smoking were associated with accelerated atherosclerosis progression as assessed by the increase in carotid artery intimal-medial thickness (Howard et al. 1998), and the risk of death due to CVD significantly increases in nonsmokers regularly exposed to environmental tobacco smoke (ETS) (Ambrose and Barua 2004; Glantz and Parmley 1995; He et al. 1999; Rosamond et al. 2007). Despite overwhelming evidence indicating that tobacco smoke is one of the most significant risk factors for CVD development, essentially little is known about the fundamental processes of how it increases CVD susceptibility.

Mitochondria are multifunctional organelles that are essential for a variety of cell functions, including energy production, thermogenesis, apoptosis, and redox signaling. Mitochondrial mutation, damage, and dysfunction have been associated with a broad spectrum of disorders, including CVD (Ballinger et al. 2000; Corral-Debrinski et al. 1991; Ferrari 1996; Wallace et al. 1992), and mitochondrial superoxide dismutase 2 (SOD2)-specific activity declines in mice exposed to ETS (Knight-Lozano et al. 2002). Additionally, overexpression of SOD2 can blunt the effects of proatherogenic factors such as tumor necrosis factor-α (TNF-α) (Manna et al. 1998; Zhang et al. 1992). Because SOD2 is essential for the regulation of mitochondrial oxidants, we hypothesized that altered endogenous mitochondrial oxidant stress can influence atherogenic susceptibility. We generated apolipoprotein E (apoE)-null, SOD2-heterozygous mice (apoE^−/ −^, SOD2^+/−^) that had decreased SOD2 protein levels and activity compared with control apoE^−/ −^ littermates (apoE^−/ −^, SOD2^+/+^). Results showed that apoE^−/ −^, SOD2^+/−^ mice had significantly increased atherosclerotic lesion formation compared with control apoE^−/ −^ littermates, in the presence or absence of ETS exposure, and these differences were independent of cholesterol levels. Further, *in vitro* studies using human vascular endothelial cells showed that decreased SOD2 activity significantly increased basal and TNF-α–induced cellular oxidant levels.

## Materials and Methods

### Mice

A colony of apoE^−/ −^, SOD2^+/−^ mice has been maintained beyond 40 generations on the C57Bl/6 background at the University of Alabama–Birmingham [for detailed description, see Supplemental Material (doi:10.1289/ehp.1002857)]. Both apoE^−/ −^, SOD2^+/−^ and apoE^−/ −^, SOD2^+/+^ (control) male siblings were used in these studies. Mice were fed chow diets (PicoLab Rodent Chow 20; Lab Diet, Brentwood, MO). Diet and water were supplied *ad libitum*. All animals were treated humanely, with regard to alleviation of suffering.

### Genotyping

The SOD2-knockout allele was screened by targeting amplification of the *hprt* minigene used to generate the knockout by using HPRTx1-2F (5′-CAGCCCTGGCGTCGTGATTAGT-3′) and HPRTx7R (5′-CGTGGGGTCCTTTTCACCAGCAA-3′), yielding a 2.3-kb product, and was further verified by the MSOD2x3R (5′-GAGCGACCTGAGTTGTAACATCTCC-3′) and HPRTx7F (5′-GACCCCACGAAGTGTTGGATATAAG-3′) primers (2.1-kb product).

### Exposures

Exposures were conducted at the Institute of Toxicology and Environmental Health inhalation facilities (University of California–Davis) in accordance with institutional guidelines. At 6 weeks of age, apoE^−/ −^ and sibling apoE^−/ −^, SOD2^+/−^ mice were exposed to filtered air or ETS (10 mg/m^3^ total suspended particulate) for 6 hr/day, 5 days a week, for 4 weeks. Control mice were exposed to filtered air only.

### Research cigarettes

The standard reference cigarette, 1R4F, was used (Davis et al. 1984). The cigarettes were formulated and manufactured in 1983 by the Tobacco and Health Research Institute (University of Kentucky, Lexington, KY). All cigarettes were stored at 4°C until needed. At least 48 hr prior to use, cigarettes were placed in a closed chamber at 23°C at 60% relative humidity.

### Tissue collection and preparation

Mice were anesthetized via intraperitoneal injection with ketamine/xylazine and exsanguinated via cardiac puncture. Tissues were collected, flash-frozen in liquid nitrogen, and stored at −80°C. Tissues were then homogenized in 1.2 mL protein homogenization buffer (phosphate-buffered saline, protease inhibitors, cysteine, sodium citrate). Aliquots (300 μL) were frozen for further analysis. Before enzyme assays, Amplex Red, and Western analysis, aliquots were sonicated and centrifuged at 13,000 rpm for 15 min at 4°C, and the supernatant was quantified.

### Atherosclerotic lesion quantification

Whole aortas were stained with oil red-O and quantified as previously described (Knight-Lozano et al. 2002). For additional details, see Supplemental Material (doi:10.1289/ehp.1002857).

### Quantitative polymerase chain reaction (qPCR) for evaluating mitochondrial DNA (mtDNA) damage

Aortic DNA extraction, QPCR conditions, and calculation of DNA lesion frequencies have been previously described (Ballinger et al. 1999; Knight-Lozano et al. 2002; Yakes and Van Houten 1997). For additional details, see Supplemental Material (doi:10.1289/ehp.1002857).

### Immunoblot analysis of SOD2, 3-nitrotyrosine (3-NT), and protein carbonyls

Aortic homogenates were prepared as described in Supplemental Material (doi:10.1289/ehp.1002857); 25 g protein was then loaded onto 12% SDS-PAGE gels, subjected to electrophoresis, and immunoblotted using commercially available antibodies [SOD2 (Research Diagnostics Inc., Concord MA); 3-NT (Calbiochem, San Diego, CA); anti-dinitrophenyl (Sigma Chemical Co., St. Louis, MO)] Protein carbonyls (oxidation) were detected by derivatizing homogenates with 10 mM 2,4-dinitrophenylhydrazine before electrophoresis. Blots were visualized using chemiluminescence of the secondary horseradish peroxidase–goat anti-rabbit IgG.

### SOD activity

SOD activity was determined using the cytochrome c reduction assay, as previously described (Flohé and Ötting 1984). For additional details, see Supplemental Material (doi:10.1289/ehp.1002857).

### Cholesterol determination

Total blood plasma cholesterol levels were assessed using a cholesterol lipoprotein profile (CLiP) apparatus, as previously described (Garber et al. 2000). For details, see Supplemental Material (doi:10.1289/ehp.1002857).

### Amplex Red assay

We used Amplex Red (Invitrogen, Carlsbad, CA) to quantify hydrogen peroxide (H_2_O_2_) levels in aortic homogenates following the manufacturer’s instructions [for details, see Supplemental Material (doi:10.1289/ehp.1002857)]. H_2_O_2_ generation was calibrated by constructing standard curves using known H_2_O_2_ concentrations.

### Mitochondrial isolation

Mitochondria were isolated using standard differential centrifugation techniques (Trounce et al. 1996). Briefly, aortic tissues were collected and homogenized in isolation buffer [210 mM mannitol, 70 mM sucrose, 1 mM EGTA, 0.5% fatty acid–free bovine serum albumin (BSA), and 5 mM HEPES, pH 7.2]. Fresh tissue was processed immediately for mitochondrial isolation, with all manipulations carried out at 1–4°C. Homogenates were centrifuged at 1,500*g* for 15 min at 4°C. The supernatant was transferred, and the first set of spin conditions was repeated two more times. After the three low-speed spins, the supernatant was subjected to a high-speed centrifugation (8,000*g*, 15 min, 4°C). The supernatant was discarded, the mitochondrial pellet was resuspended in isolation buffer, and the high-speed spin was repeated. Finally, the supernatant was discarded, and pellets were used immediately or stored at −80°C. For quantification of mitochondrial protein, an aliquot was removed, centrifuged at 8,000*g* for 15 min, washed in BSA-free isolation buffer, pelleted, and used for determination of protein concentration.

### Aconitase activity

Aconitase activity was determined in aortic mitochondrial isolates (two aortas per isolate) as described by Knight-Lozano et al. (2002).

### TNF-α enzyme-linked immunosorbent assay (ELISA)

Aortic homogenate levels of TNF-α were measured using the mouse TNF-α ELISA kit (Endogen, Cambridge, MA) according to manufacturer’s instructions. For a detailed description, see Supplemental Material, page 6 (doi:10.1289/ehp.1002857).

### Small interfering RNA (siRNA) transfection

Silencer siRNA was custom designed for SOD2 (Qiagen, Valencia, CA) and used for treatment of confluent human umbilical vein endothelial cell (HUVEC) monolayers for passages 3–5 [see Supplemental Material (doi:10.1289/ehp.1002857)]. Cells were grown in endothelial cell basal media (EBM; Lonza, Walkersville, MD) Pecam-1 antibody was from Santa Cruz Biotechnology Inc, (Santa Cruz, CA). Control HUVECs were treated with siRNA-negative control or received no treatment. Twenty-four hours after siRNA treatment, cultures were treated with 30 ng/mL TNF-α for 30 min. Cells were then visualized using fluorescence microscopy or lysed for further experiments. A total of three independent experiments were performed for each treatment.

### Rhodamine 123 fluorescence

HUVECs were subsequently treated with dihydrorhodamine 123 (Molecular Probes; Invitrogen) at a final concentration of 10 μM for 30 min at 37°C in 5% carbon dioxide, rinsed with Earle’s balanced salt solution, and immediately analyzed (excitation, 485 nm; emission, 530 nm). Fluorescence intensity was quantified using ImageJ software (version 1.41; National Institutes of Health 2010) following the software instructions.

### Statistical analysis

A total of five animals per group were used for each end point, except for *in vitro* studies (three independent experiments). Results are expressed as mean ± SE. We used analysis of variance to test the null hypothesis that all samples were drawn from a single population. If significant differences (*p* < 0.05) existed, we used a Student-Newman-Keuls test for group comparisons.

## Results

To determine whether compromised mitochondrial function and oxidative stress can influence atherogenic susceptibility, we generated apoE^−/ −^ mice with decreased SOD2 protein and activity levels via crosses with SOD2^+/−^ mice, and backcrossed these offspring to apoE^−/ −^ mice to yield apoE^−/ −^, SOD2^+/−^ animals. We observed no significant differences in CuZn (copper-zinc) SOD activity, consistent with previous studies on SOD2^+/−^ mice (Van Remmen et al. 1999). Aortic SOD2 protein levels were significantly decreased in apoE^−/ −^, SOD2^+/−^ mice compared with apoE^−/ −^, SOD2^+/+^ (apoE^−/ −^) littermates, and this decrease corresponded to a significant decrease in aortic SOD2 activity compared with control apoE^−/ −^ littermates [see Supplemental Material, Figure 1 (doi:10.1289/ehp.1002857)].

**Figure 1:**
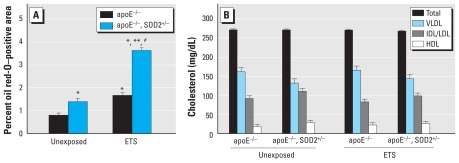
Assessment of atherosclerotic lesions (aortic oil red-O staining) in apoE^−/ −^ and apoE^−/ −^, SOD2^+/−^ mice exposed to filtered air or ETS. (*A*) Quantification. (*B*) Plasma lipoprotein profiles. Abbreviations: HDL, high-density lipoprotein; IDL/LDL, intermediate-/low-density lipoprotein; VLDL, very low-density lipoprotein. *p < 0.05 compared with unexposed apoE^−/ −^ mice. **p < 0.05 compared with exposed apoE^−/ −^ mice. ^#^*p* < 0.05 compared with unexposed apoE^−/ −^, SOD2^+/−^ mice.

We then quantified the impact of decreased SOD2 activity on atherogenesis by oil red-O staining of whole aortas harvested from mice exposed to either filtered air or ETS. apoE^−/ −^ mice with decreased SOD2 activity had significantly higher oil red-O staining compared with apoE^−/ −^ littermates in both the unexposed and ETS-exposed groups [Figure 1A; see also Supplemental Material, Figure 2 (doi:10.1289/ehp.1002857)]. These effects were not associated with differences in cholesterol levels between or among genotypes or exposure groups (Figure 1B), consistent with the notion that elevated mitochondrial oxidative stress can increase individual susceptibility to atherogenic factors.

**Figure 2:**
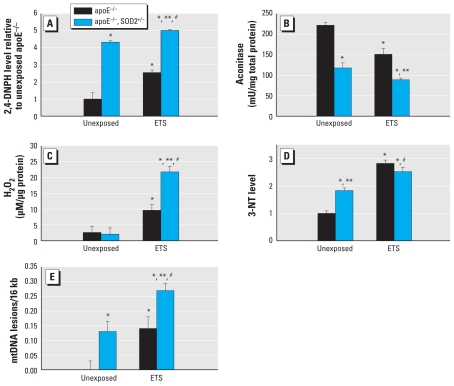
Cellular and mitochondrial oxidative stress levels in apoE^−/ −^ and apoE^−/ −^, SOD2^+/−^ mice exposed to filtered air or ETS. Quantification of oxidized protein [2,4-dinitrophenylhydrazine (2,4-DNPH)] (*A*), aconitase activity (*B*), H_2_O_2_ (*C*), 3-NT (*D*), and mtDNA damage (*E*) in mice exposed to filtered air or ETS. *p* < 0.05 compared with unexposed apoE^−/ −^ mice. **p < 0.05 compared with exposed apoE^−/ −^ mice. ^#^*p* < 0.05 compared with unexposed apoE^−/ −^, SOD2^+/−^ mice.

To determine whether decreased SOD2 activity increased general levels of cellular oxidants, we examined oxidized protein levels from aortic homogenates prepared from apoE^−/ −^, SOD2^+/−^ mice and apoE^−/ −^ littermate controls. apoE^−/ −^, SOD2^+/−^ mice had significantly higher levels of oxidized proteins than did exposure-matched apoE^−/ −^ littermates (Figure 2A). To assess mitochondrial oxidant load, we quantified aconitase activity from mitochondrial isolates [see Supplemental Material, page 7 (doi:10.1289/ehp.1002857)]. Aconitase is specifically inactivated by superoxide (O_2_^•−^) and peroxynitrite (ONOO^−^). Figure 2B shows a significant decrease in mitochondrial aconitase activity in apoE^−/ −^, SOD2^+/−^ mice compared with exposure-matched apoE^−/ −^ littermates, which was further decreased by ETS exposure. Measurement of H_2_O_2_ levels in both genotypes indicated that no differences existed between apoE^−/ −^ and apoE^−/ −^, SOD2^+/−^ mice exposed to filtered air (Figure 2C); however, ETS exposure significantly increased levels in both genotypes compared with unexposed controls and elicited the highest levels of H_2_O_2_ in the apoE^−/ −^, SOD2^+/−^ mice. Interestingly, we observed a contrasting relationship when determining the degree of reactive nitrogen species formation using 3-NT levels. Unexposed apoE^−/ −^, SOD2^+/−^ mice had higher levels of 3-NT than matched apoE^−/ −^ animals (Figure 2D); however, we observed no differences between groups exposed to ETS, although levels of 3-NT were increased relative to unexposed controls. We assessed mtDNA damage (Figure 2E) and found increased mtDNA damage in apoE^−/ −^, SOD2^+/−^ mice compared with exposure-matched controls.

**Figure 3:**
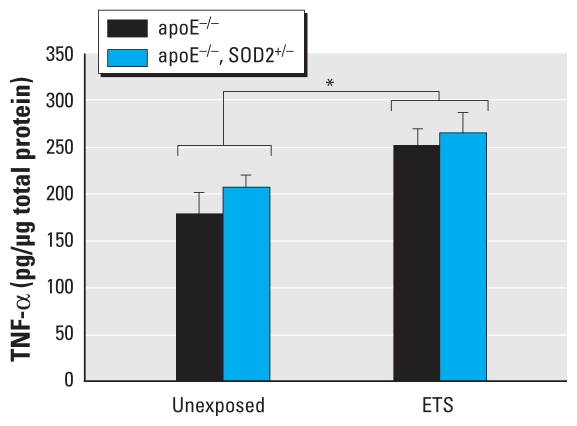
TNF-α levels in aortic tissue homogenates from apoE^−/ −^ and apoE^−/ −^, SOD2^+/−^ mice exposed to filtered air or ETS (*n* = 5 per group). TNF-α protein levels were measured at an absorbance of 450 nm and calculated in pg/μg total protein, determined from a standard curve. *p < 0.05 between exposure groups.

**Figure 4:**
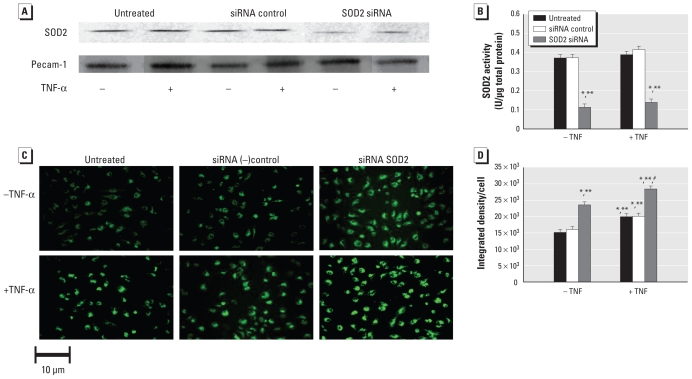
TNF-α–induced oxidant production in HUVECs pretreated with sham (untreated), control (siRNA ctl), or SOD2 siRNA for 24 hr, followed by no treatment or by treatment with 30 ng/mL TNF-α for 30 min. (*A*) SOD2 and platelet-endothelial cell adhesion molecule (Pecam-1) levels. (*B*) SOD2 activity. Assessment (*C*) and quantification (*D*) of rhodamine 123 fluorescence. *p < 0.05 compared with untreated cells. **p < 0.05 compared with siRNA controls. ^#^*p* < 0.05 compared with all other groups.

TNF-α has been implicated as an important proatherogenic factor (Churg et al. 2002), and it is also known to mediate its cellular effects via mitochondrial oxidant generation (Meier et al. 1989; Satriano et al. 1993; Schulze-Osthoff et al. 1992); therefore, the effects of decreased SOD2 activity (as in our apoE^−/ −^, SOD2^+/−^ mice) on TNF-α levels were of interest in these studies. Although TNF-α protein in aortic tissues increased with ETS exposure, they did not significantly differ between genotypes (apoE^−/ −^, SOD2^+/−^ vs. apoE^−/ −^ mice) (Figure 3). Consequently, to determine the impact of decreased SOD2 activity on endothelial response to the same “dose” of TNF-α, we assessed the effects of TNF-α–induced oxidant production in HUVECs in which SOD2 levels and activity were decreased by siRNA transfection. Decreased SOD2 activity increased endothelial cell oxidant levels in the absence or presence of TNF-α compared with HUVEC controls (Figure 4), consistent with the notion that decreased mitochondrial antioxidant capacity can alter cellular response to inflammatory cytokines such as TNF-α.

## Discussion

Our findings are consistent with the hypothesis that endogenous mitochondrial oxidant stress can influence the impact of CVD risk factors. Decreased SOD2 activity increased measures of cellular and mitochondrial oxidant stress and, moreover, atherosclerotic lesion development under conditions of hypercholesterolemia and ETS exposure. These effects were not associated with changes in cholesterol levels between genotypes or exposure regimens and hence demonstrate that decreased mitochondrial antioxidant capacity can increase susceptibility to CVD development. Similarly, decreased SOD2 activity in endothelial cells increased both basal and TNF-α–mediated cellular reactive oxygen species (ROS) levels, suggesting that mitochondrial antioxidant capacity affects cellular response to inflammatory factors.

Of the three isoforms of SOD, only SOD2 is essential for life. Mice null for cytosolic SOD1 and extracellular SOD3 are viable (Matzuk et al. 1998), whereas SOD2^−/ −^ mice succumb to cardiac and/or central nervous system failure as neonates (Lebovitz et al. 1996). This fact emphasizes the importance of the mitochondrion and the regulation of its oxidant levels for proper cellular function. Studies have shown that compromised SOD2 activity influences vessel relaxation (Chen et al. 2007), endothelial nitric oxide synthetase expression (Chen et al. 2007), hypertension (Rodríguez-Iturbe et al. 2002), and recovery from ischemia reperfusion (Asimakis et al. 2002). Similarly, increased SOD2 expression has been shown to be protective against ischemia reperfusion injury, xenobiotic cardiotoxicity, alcohol-induced liver injury, and diabetic cardiomyopathy (Chen et al. 1998; Shen et al. 2006; Wheeler et al. 2001; Yen et al. 1999). In the present study, decreased SOD2 protein levels and activity significantly increased mitochondrial damage, protein modification, and oil red-O staining areas in apoE^−/ −^, SOD2^+/−^ mice. Although these effects were higher with ETS exposure, even unexposed apoE^−/ −^, SOD2^+/−^ mice generally had higher levels of oxidants/damage than did control apoE^−/ −^ littermates. Overall, these findings suggest that mitochondrial oxidative stress and damage play an important role in influencing susceptibility to CVD risk factors.

Although ETS exposure caused a significant decrease in SOD2 activity in apoE^−/ −^ mice compared with unexposed controls, we observed no significant differences between the apoE^−/ −^, SOD2^+/−^ exposure groups [see Supplemental Material, Figure 1 (doi:10.1289/ehp.1002857)]. This may be due to potential “threshold” effects associated with decreased SOD2 activity. Cells with SOD2 activities that fall below a minimal threshold required for survival would be lost, leaving those with sufficient SOD2 activity as survivors. Studies showing that compromised SOD2 activity increases sensitivity to cell death are consistent with this concept (Fujimura et al. 1999; Kokoszka et al. 2001; Strassburger et al. 2005; Van Remmen et al. 2001).

ETS exposure increased both H_2_O_2_ and 3-NT levels relative to unexposed, genotype-matched controls. However, no differences in H_2_O_2_ levels existed between unexposed apoE^−/ −^ and apoE^−/ −^, SOD2^+/−^ mice, whereas ETS exposure significantly increased H_2_O_2_ levels in apoE^−/ −^, SOD2^+/−^ mice relative to exposed apoE^−/ −^ (Figure 2C). In contrast, 3-NT levels were significantly different in unexposed animals (apoE^−/ −^ vs. apoE^−/ −^, SOD2^+/−^), and although ETS exposure increased 3-NT levels in both genotypes compared with their unexposed counterparts, we found no differences in 3-NT levels between exposed apoE^−/ −^ and apoE^−/ −^, SOD2^+/−^ mice (Figure 2D). These data suggest that in the unexposed animals, apoE^−/ −^, SOD2^+/−^ mice tend to generate more reactive species involving nitric oxide (^•^NO) pathways compared with apoE^−/ −^ (Figure 2D), whereas conditions associated with ETS exposure appear to favor pathways contributing to H_2_O_2_ production in the apoE^−/ −^, SOD2^+/−^ mouse (Figure 2C). Although apoE^−/ −^, SOD2^+/−^ mice generate greater levels of O_2_^•−^, ^•^NO concentrations are likely higher than O_2_^•−^ levels in the vasculature under unexposed conditions; furthermore, because the rate constant for O_2_^•−^ reaction with ^•^NO is substantially higher than that for SOD2 (Beckman and Koppenol 1996), ONOO^−^ formation is favored, which contributes to both protein oxidation (Figure 2A) and, in the presence of CO_2_, nitration reactions [ONOO^−^ reacts with CO_2_ to form peroxynitrosopercarbonate (ONOOCO_2_^−^), a nitrating agent; Figure 2D] (Denicola et al. 1996). ETS exposure further enhances mitochondrial O_2_^•−^ production in both genotypes, and as O_2_^•−^ levels rise, oxidative reactions would become more frequent as ^•^NO/O_2_^•−^ ratios decrease (Jourd’heuil et al. 2001). Because apoE^−/ −^, SOD2^+/−^ mice generate higher levels of O_2_^•−^, the ^•^NO/O_2_^•−^ ratio in these animals would be lower, shifting reaction of O_2_^•−^ toward H_2_O_2_ production via spontaneous or enzymatically mediated dismutation of O_2_^•−^. Additionally, SOD2^+/−^ mice have significantly decreased levels of reduced glutathione (Van Remmen et al. 1999), which would further contribute to cellular H_2_O_2_ levels. The enhancement of ^•^NO-mediated actions by SOD addition and the association of decreased SOD2 activity with decreased vessel relaxation are consistent with the notion of altered ^•^NO bioavailability in SOD2-deficient mice (Chen et al. 2007). Increased mitochondrial oxidant generation may also trigger increased nonmitochondrial sources of H_2_O_2_ production when challenged with ETS. These findings suggest that the species contributing to cellular stress can vary, depending upon endogenous and exogenous factors. Because both protein oxidation and mtDNA damage can be mediated by several different reactive species, the results presented in Figure 2A and E are not inconsistent with this concept.

A key element in the atherogenic cascade is an increase in local inflammatory cytokines, such as TNF-α, which is a pleiotrophic proinflammatory cytokine produced by macrophages, endothelial cells, and smooth muscle cells with a wide variety of biological activities that affect important components of atherosclerotic lesion development (de Frutos et al. 2001; Gao et al. 2007; Morita et al. 1995). TNF-α exerts potent proatherogenic effects such as increased leukocyte rolling, adhesion, and emigration by induction of intercellular adhesion molecules and vascular endothelial cell adhesion molecules (Maekawa et al. 2009; Morita et al. 1995). Correspondingly, TNF-α–null, apoE^−/ −^ mice have significantly decreased atherosclerotic lesion development compared with control apoE^−/ −^ mice, consistent with the notion that TNF-α plays an important role in atherogenesis (Brånén et al. 2004; Ohta et al. 2005). Finally, several reports have shown that TNF-α mediates mitochondrial oxidant stress and that decreased SOD2 activity influences cellular sensitivity to TNF-α–mediated apoptosis (Bruce-Keller et al. 1999; Kim and Kim 1999; Manna et al. 1998; Suzuki et al. 1997). Studies have also shown that overexpression of SOD2 suppresses TNF-α–induced apoptosis and activation of nuclear factor κB (NF-κB) signaling (Manna et al. 1998). Consequently, mitochondrial ROS production in response to cellular stimuli likely affects NF-κB signaling pathways, potentially via activation of upstream redox-sensitive kinases [I κB kinase (IKK) complex or NF-κB kinase] that are important in NF-κB signaling. Because our results showed no significant differences in TNF-α levels between genotypes, yet oxidant stress was significantly higher in apoE^−/ −^, SOD2^+/−^ mice, we hypothesized that the same amount of TNF-α would increase cellular oxidant production in endothelial cells having decreased SOD2 activity compared with controls. In fact, our *in vitro* results support this notion (Figure 4). Consequently, an extension of this concept is that decreased mitochondrial function and integrity influenced by age and/or exposure to CVD risk factors enhance the impact of proatherogenic factors such as TNF-α in the endothelium.

**Figure 5:**
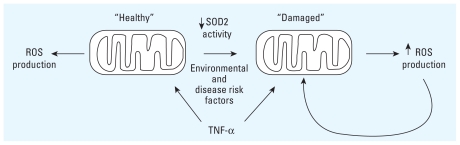
“Healthy” mitochondrial response to cellular stimuli such as TNF-α generates functional parameters that result in normal cell function. Disease risk factors cause mitochondrial damage that changes cellular response stimuli via increased oxidants, which creates a cycle of mitochondrial ROS production and inflammation.

We therefore propose that mitochondrial integrity and function are important in determining individual vulnerability to common CVD risk factors such as ETS or hypercholesterolemia. Sustained mitochondrial damage (due to CVD risk factor exposure, age, etc.) influences the way cells respond to cellular stimuli by altering mitochondrial energetics and oxidant regulation. Hence, the same “dose” of a cellular stimulus, such as TNF-α (Figure 5), can produce significantly different effects between cells with “healthy” or undamaged versus damaged mitochondria. In this model, mitochondria that sustain damage via aging or environmental stressors will generate increased oxidants in response to the same “dose” of a stimuli and therefore are more prone to activate redox-sensitive kinases (e.g., IKK complex or NF-κB kinase), which in turn activate signaling cascades that can be proinflammatory. Resultant cytokines would further induce mitochondrial ROS production, creating a cycle of inflammation and mitochondrial ROS production.

Because mitochondrial function and integrity decline over time and are influenced by changes in the cellular environment (e.g., stress induced by behavioral and/or environmental factors), this model provides a means for explaining the increasing risk of disease development associated with age and exposure to known risk factors. Finally, because “normal” mitochondrial and nuclear genetic variation that occurs in humans likely influences mitochondrial functional characteristics, individual variation in CVD susceptibility may be based, in part, upon differences in mitochondrial function and susceptibility to mitochondrial damage. This mitochondrial response concept is complementary with the inflammatory response theory in that the accumulation of oxidative stress associated with inflammation causes mitochondrial damage in vascular tissues, which may further enhance the proinflammatory pathways that lead to vascular cell dysfunction, a key step in the disease development.
